# Effectiveness of malic acid 1% in patients with xerostomia induced by antihypertensive drugs

**DOI:** 10.4317/medoral.18206

**Published:** 2012-08-28

**Authors:** Gerardo Gómez-Moreno, Javier Guardia, Antonio Aguilar-Salvatierra, Maribel Cabrera-Ayala, José E. Maté-Sánchez de-Val, José L. Calvo-Guirado

**Affiliations:** 1 Senior Lecturer of Special Care in Dentistry. Professor responsible of Pharmacological Interactions in Dentistry. Faculty of Dentistry, University of Granada, Granada, Spain; 2 Doctor in Dentistry. Collaborator of Pharmacological Interactions in Dentistry. Faculty of Dentistry, University of Granada, Granada, Spain; 3 Degree in Dentistry. Collaborator of Pharmacological Interactions in Dentistry. Faculty of Dentistry, University of Granada, Granada, Spain; 4 Associate Professor Restorative Dentistry. Faculty of Medicine and Dentistry, University of Murcia, Murcia, Spain; 5 Senior Lecturer of General and Implant Dentistry. Faculty of Medicine and Dentistry, University of Murcia, Murcia, Spain

## Abstract

Objectives: Assessing the clinical effectiveness of a topical sialogogue on spray (malic acid, 1%) in the treatment of xerostomia induced by antihypertensive drugs. Study Design: This research has been carried out through a randomized double-blind clinical trial. 45 patients suffering from hypertensive drugs-induced xerostomia were divided into 2 groups: the first group (25 patients) received a topical sialogogue on spray (malic acid, 1%) whereas the second group (20 patients) received a placebo. Both of them were administered on demand for 2 weeks. Dry Mouth Questionnaire (DMQ) was used in order to evaluate xerostomia levels before and after product/placebo application. Unstimulated and stimulated salivary flows rates, before and after application, were measured. All the statistical analyses were performed by using SPSS software v17.0. Different DMQ scores at the earliest and final stage of the trial were analysed by using Mann-Whitney U test, whereas Student’s T-test was used to analyse salivary flows. Critical p-value was established at p<0.05. Results: DMQ scores increased significantly (clinical recovery) from 1.21 to 3.36 points (p<0.05) after malic acid (1%) application whereas DMQ scores increased from 1.18 to 1.34 points (p>0.05) after placebo application. After two weeks of treatment with malic acid, unstimulated salivary flow increased from 0.17 to 0.242 mL/min whereas the stimulated one increased from 0.66 to 0.92 mL/min (p<0.05). After placebo application unstimulated flow ranged from 0.152 to 0.146 mL/min and stimulated flow increased from 0.67 to 0.70 mL/min (p>0.05). Conclusions: Malic acid 1% spray improved antihypertensive-induced xerostomia and stimulated the production of saliva.

** Key words:**Xerostomia, hyposialia, malic acid, antihypertensive drugs.

## Introduction

Xerostomia is a subjective complaint of dry mouth, and it is usually caused by a decreased salivary flow or by changes in the biochemical composition of saliva. Patients suffering from xerostomia usually complain about difficulties when chewing, swallowing or even speaking, particularly those with dental prosthesis. Whereas xerostomia is a subjective concept, hyposalivation makes reference to a decreased salivary flow and it is, therefore, an objective and measurable variable (1-3).

Hyposalivation is considered to appear when salivary flow rates are under 0.1-0.2 mL/min (unstimulated) or 0.7 mL/min (stimulated). Xerostomia is often associated with hyposalivation, but not always. It is widely accepted that a salivary flow rate reduction of around 50% implies the manifestation of signs and symptoms of xerostomia (4,5). However, some cases have been described in patients with a normal salivary flow rate. Furthermore, other patients with a decreased salivary flow rates (generally fewer than 50% if compared to normal levels) have not complaint about oral problems.

The most important aetiological factors related to xerostomia are: head and neck radiotherapy, some systemic conditions (primary or secondary Sjögren syndrome, stress, diabetes, clinical depression) and also the intake of certain drugs (1,6,7). More than 500 drugs (8), including 42 different pharmacological groups (9) can cause xerostomia as a side effect. Drugs with the most intense xerostomizing effect are: -those with a direct impact on the autonomic nervous system, which regulates gland secretions (anticholinergic agents; adrenergic blockers); - those with an indirect impact on the central nervous system (antipsychotic drugs, anxiolytic agents); - those which increase the excretion of liquids, particularly diuretics. In addition to this, some psychological factors, such as stress, anxiety or depressive conditions are also related to xerostomia. However, hiposalivation is normally associated with drug-induced xerostomia, whereas this association is not usual in the case of xerostomia related to psychological conditions (10).

Drugs most commonly associated with xerostomia are: -antidepressants (particularly tricyclic antidepressants) (11); - Selective Serotonin Reuptake Inhibitors (SSRIs), particularly when combined with benzodiazepines (12); -Diuretics, antihypertensive drugs and angiotensin-converting enzyme inhibitors (ACE inhibitors), -oral hypoglycemiants,-acetylsalicylic acid (ASA), -iron supplements. Let us not forget that drugs with the most intense xerostomizing effect are also the most widely and frequently used (treatment of metal disorders and cardiovascular diseases) (13). This fact is useful to explain the important prevalence among adults older than 65 (20-46%) (6,14,15). Such prevalence is caused by 3 important factors: accumulation of systemic conditions, polymedication and the progressive decline of salivary gland parenchyma.

There is a wide range of therapies in the treatment of xerostomia (sialogogues, salivary substitutes, general measures…), although the efficiency of many of them is controversial (1,6,7). Abundant literature has described malic and citric acid as salivary stimu-lants, although they were dropped out because their demineralizing effect on the tooth enamel (16,17). Nevertheless, recent researches have reported a decrease of this demineralizing potential of malic acid, if used at an appropriate concentration (4.7%) and combined with xylitol and fluorides (18).

Assessing the clinical efficiency of a topical sialogogue on spray (malic acid 1%), combined with xylitol and fluoride, in the treatment of xerostomia induced by antihypertensive drugs for 2 weeks has been the main objective of this research.

## Material and Methods

-Patients recruitment and intervention 

This randomized double-blind clinical trial has been approved by the University of Granada Ethics Committee (Spain). Randomization was done following the guidelines of The Consort Statement (http://www.consort-statement.org/consort-statement/). The sample size calculation was performed from the standard deviation of the main variable (DMQ). 45 participants with xerostomia caused by the chronic admnistration of antihypertensive drugs, were recruited at the Faculty of Dentistry of the University of Granada (Spain) and also at the Faculty of Medicine and Dentistry of the University of Murcia (Spain). No participants left the trial.

-Inclusion Criteria

-Subjects under antihypertensive treatment (longer than one month) with xerostomia.

-Exclusion Criteria

-History of head and neck radiotherapy, Sjögren’s syndrome and related autoimmune diseases (rheumatoid arthritis, polyarthritis nodosa, systemic sclerosis or lupus erythematosus).

-Subjects with diabetes and/or oral hypoglycemic therapy.

-Chronic alcoholic subjects

The 45 patients suffering from hypertensive drugs-induced xerostomia were divided into 2 groups: the first group (25 patients) received a topical sialogogue on spray (malic acid, 1%) whereas the second group (20 patients) received a placebo. 16 patients were being treated with diuretics, 14 with angiotensin-converting enzyme inhibitors (ACE inhibitors), 7 with non-selective beta-blockers, and 8 with angiotensin II receptor antagonists.

The randomized distribution was designed by using the websitehttp://www.randomization.com, and obtaining a randomization plan, which assigned participants to a “test group” (to be treated with 1% malic acid on spray) or to a “placebo group” (to be treated with a spray with no malic acid). This randomization plan was delivered to a person not related to the project in order to prevent both participants and observers from identifying the product.

Once the patients had signed the informed consent form and anamnesis had been performed, the following question was asked to every patient: “How often do you feel dry mouth?” Available answers were: “never”, “sometimes”, “usually” or “always”. Those who answered “usually” or “always” were considered as suffering from xerostomia.

Our clinical intervention was based on the application of a topical sialogogue, containing 1% malic acid (Xeros Dentaid spray©, Dentaid, Barcelona, Spain) for 2 weeks in an experimental group of 25 patients, whereas a control group of 20 patients was given a placebo with the same presentation and composition (excepting malic acid). Product/placebo was presented without any brand name, and they were administered on demand, with a maximum of 8 doses per day.

Dry Mouth Questionnaire (DMQ)

DMQ, developed by Vissink et al. (19), Gravenmade et al. (20), van der Reijden et al. (21) y Regelink et al. (22) (Table 1), was used in order to obtain subjective information about the severity of xerostomia before and after treatment with malic acid/placebo. Every participant had to answer an initial questionnaire (DMQ 1) about the symptoms related to oral dryness, and received a spray (malic acid or placebo). After 2 weeks of treatment, patients had to answer again DMQ 1, and also a new questionnaire (DMQ 2) about the efficiency of the treatment.

DMQ 1 was used to assess the initial severity of oral dryness, particularly its impact on oral function: problems when chewing, swallowing, speaking and impact on daily life.

DMQ 1 used a 0-to-4 rating scale where 0 = “very dry” and 4 = “not dry at all”. After 2 weeks of treatment, DMQ 1 was repeated ( Table 1). On the other hand, DMQ 2 was designed to assess the impact of the spray on the symptoms of xerostomia, and was also based on a 0-to-4 rating scale where 0 = frequent restriction of oral function and 4= no restriction of oral function/ no oral dryness feeling. Application frequency and retention time inside the oral cavity were also registered ( Table 1).

**Table 1:**
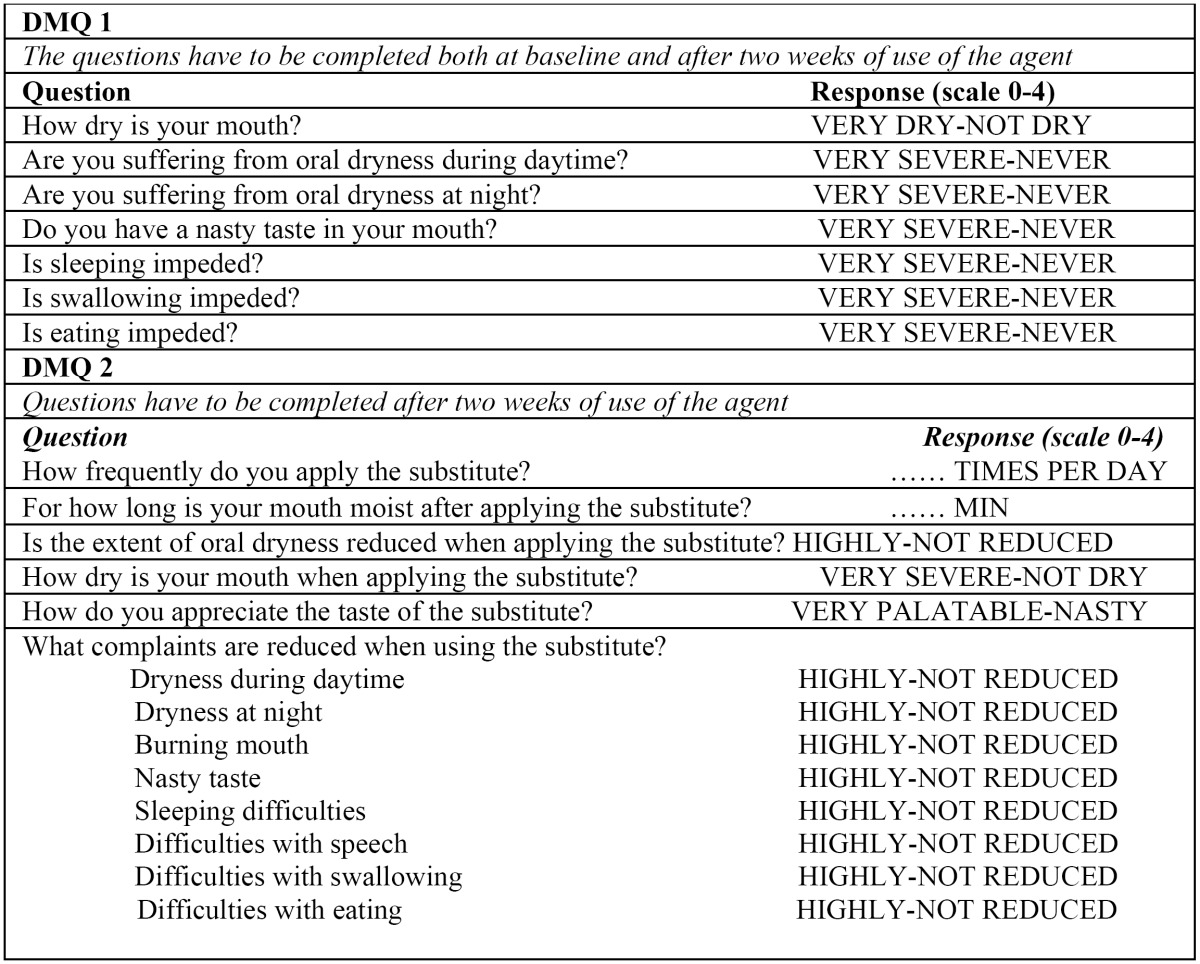
Dry Mouth Questionnaire (DMQ).

-Sialometries

As secondary measures, both unstimulated and stimulated salivary flow rates were assessed in all patients. Unstimulated salivary flow rate was obtained through the collection of saliva every 30 seconds during 15 minutes. Saliva was collected on 20mL plastic containers, which were pre-weighted (0.001 g) by using a precision scale (Cobos M-150, Cobos, Barcelona, Spain). Measures were expressed as mL/min. As far as stimulated flow rate is concerned, it was obtained by chewing a 1 g piece of paraffin during 6 minutes. Saliva collected during the first minute was discarded, and then collected on containers every 30 seconds. DMQ and sialometries were always performed from 09:00 a.m. to 11:00 a.m. in order to avoid any circadian variation. Prior to intervention, patients were told not to eat, drink, smoke or brush their teeth from one hour before their visit.

-Statistical analysis

All of the analyses were performed by using SPSS software v17.0 (SPSS Inc. Chicago, IL, USA). The main purpose was to contrast different DMQ scores at the earliest and final stage of the clinical trial by using the Mann-Whitney U test. Student’s T-test was used to analyse both unstimulated and stimulated salivary flow rates. Critical p-value was established at p<0.05.

## Results

 Table 2 shows the results of our clinical trial in relation to age, gender, DMQ score (oral dryness feeling), number of applications and duration of sialogogue action. No relevant statistical differences were found as far as gender is concerned. DMQ scores related to dry mouth feeling increased significantly (therefore suggesting a clinical recovery) from 1.21 ± 0.14 points to 3.36 ± 0.17 points (p<0.05) after two weeks treatment with malic acid, whereas in the control group DMQ scores increased from 1.18 ± 0.12 points to 1.34 ± 0.09 points (p>0.05). 92% of the patients treated with malic acid experienced some clinical recovery, in contrast to just 15% of the patients treated with placebo. Moreover, no patient in the experimental group experienced a decline of the initial condition, whereas 3 members of the control group did it. Patients belonging to the experimental group used the product 3.71± 1.32 times per day, whereas those belonging to the control group used the placebo 6.75 ± 1.21 times per day.

**Table 2:**
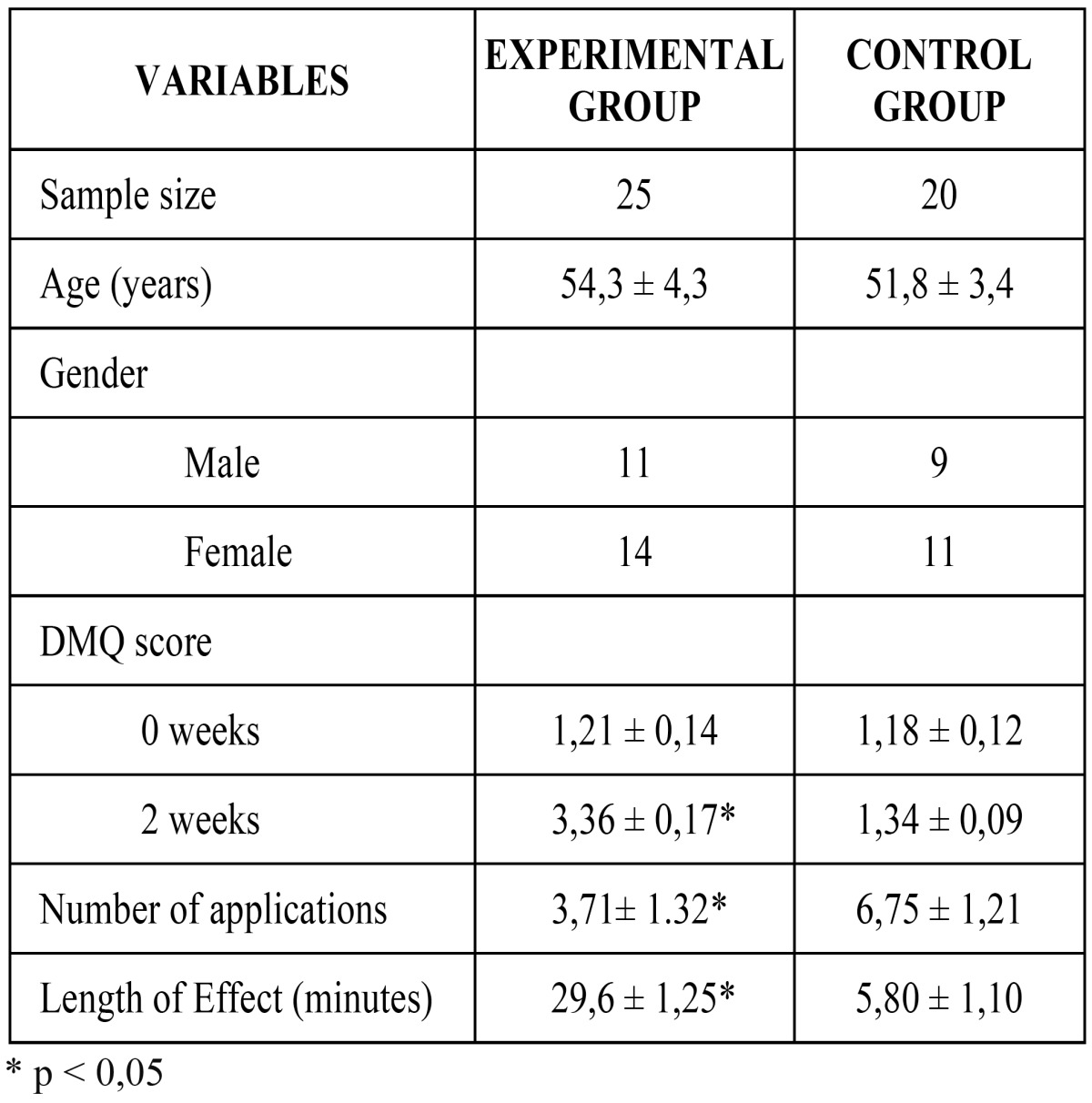
Age, gender, DMQ score (severity of xerostomia) and length of effect of the participants. Average ± standard deviation.

**Figure 1:**
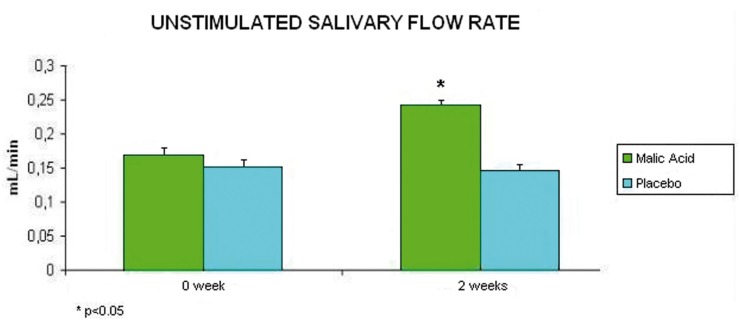
Average and standard deviation of unstimulated salivary flow rates of the participants (at the beginning and after two weeks of treatment).

**Figure 2:**
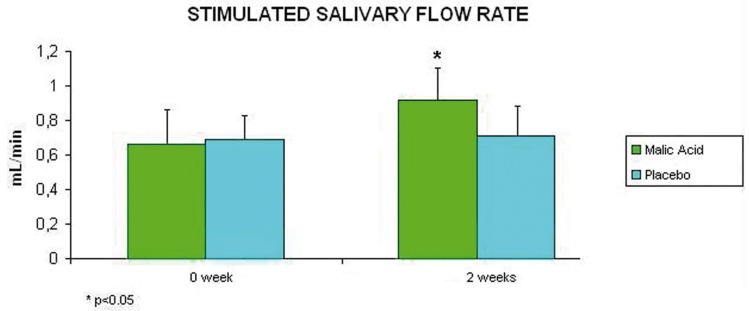
Average and standar deviation of stimulated salivary flow rates of the participants (at the beginning and after two weeks of treatment).

Regarding salivary flow rates, they improved after treatment with 1% malic acid. After 2 weeks of treatment with the product, unstimulated salivary flow rate increased significantly from 0.170 mL/min to 0.242 mL/min (p<0.05), whereas patients treated with placebo ranged from 0.152 mL/min to 0.146 mL/min (p>0.05) (Fig. 1). As far as stimulated salivary flow rates are con-cerned, patients treated with malic acid experienced a significant average increase from 0.660 mL/min to 0.920 mL/min after 2 weeks treatment (p<0.05); whereas those patients treated with placebo experienced an average increase from 0.67 mL/min to 0.70 mL/min after 2 weeks (p>0.05) (Fig. 2).

## Discussion

The most important aetiological factor related to xerostomia is the intake of drugs. The interruption of the treatment or substitution of these drugs could increase the salivary flow rate to the level previous to treatment. Nevertheless, this practice involves a risk for the health of the patient, as it would improve oral dryness but would also aggravate the previous condition (in our research, this previous condition is arterial hypertension).

Xerostomia induced by hypertensive drugs is reversible, since even with this condition the salivary glands are sufficiently functional. Consequently, they can be treated with salivary stimulants (sialogogues). Treatment with systemic sialogogues with anticholinesterasic and cholinergic action represents an efficient therapeutic option, although they are usually dropped out because of the quantitative and qualitative importance of their side effects. In this context, the application of topical sialogogues can be a useful alternative in the treatment of reversible xerostomia induced by drugs, as in spite of the fact that their therapeutic effects are more transitory and less lengthy; their side effects are also less powerful (23).

The application of topical sialogogues containing acids in the treatment of xerostomia is not recent. However, continuous application of substances such as citric acid (24) has been related to an increased risk of caries, as a consequence of the erosive action of these agents over the dentin. Similarly, the use of chewing gums containing 0.06 mg of ascorbic or malic acid combined with important quantities of sorbitol and mannitol (16) can cause similar negative effects.

All of these products were dropped out because of their demineralising effect on the human dentin, effect not only caused by the high doses of acidic products, but also by the product presentation (chewable products), which allows a lengthy contact with the dental surfaces. In order to solve this, presentation in spray format allows a fast and direct contact with the oral mucosa, and this fact, if combined with a suitable concentration (as the stimulant effect on saliva production is not altered by it), could reduce the demineralising potential of these substances (25,26,18).

According to the researches carried out by da Mata et al. (18) the use of 4.7% malic acid on spray (combined with fluorides and xylitol), on 60 healthy subjects induced a significant drop of salivary pH levels, which recovered 20 minutes later. Nevertheless, when this acidic salivary stimulant contains xylitol/fluorides, the subsequent decrease in the salivary pH level never reaches a score lower than 5.5 (hydroxyapatite critical level) (18). Thus, combination of malic acid with xylitol/fluorides on spray seems to be a safe option as topical sialogogue (9). According to the results of our clinical research, the use of malic acid as a salivary stimulant, if combined with xylitol and fluorides, can be a valid option in the treatment of mild and reversible xerostomia induced by antihypertensive drugs. Malic acid acts as a substance capable of generating a sour taste gustatory stimulus. Its mechanism of action is linked to dissociation of malic acid in H+, which they join water to become hydronium ions (H3O+); this action generates a stimulation of salivary secretion to dilute the concentration of acids in the oral cavity. Xylitol and fluorides do not stimulate saliva but they reduce erosion and cariogenic potential.

When used as a topical sialogogue, this product provides a short term effect increasing salivary flow rates immediately and subjects in this clinical trial reported a feeling of increase saliva production for an average period of 29 minutes (in contrast to an average period of 5 minutes in the control group). These results are in line with the results obtained by da Mata et al. (18).

Salivary flow rates became normal 20-30 minutes after application, and patients did not feel the need for a new application for around 6 hours time, so 3 or 4 applications per day were enough. In contrast, patients treated with placebo used it 6 or 7 times per day.

Among the different available questionnaires to evaluate the severity of oral dryness, we chose the DMQ (19-22), since it is an easy and fast method to assess the efficiency of the product. In addition to this, its 0-to-4 scale can be easily replaced by a Visual Analogue Scale (VAS) of 10 cm. Therefore, DMQ has been a valid and useful tool for the carrying out of our clinical trial.

The results of our randomized clinical trial clearly show a significant increase of salivary flow rates, both unstimulated and stimulated, after the treatment with 1% malic acid on spray. These results are in accordance with those obtained by da Mata et al. (18), although we must point out that in that research, the subjects were healthy individuals who were not suffering from xerostomia. No additional researches or studies have focused on the assessment of the efficiency of malic acid in the treatment of xerostomia, so our clinical trial is one of the first ones dealing with this topic.

As far as subjective improvement of xerostomia is concerned, there are not similar papers to compare the results of our trial, as research in this field has been neglected because of the caries-inducing role of acidic substances when they are not properly formulated. In this sense, our clinical trial is a pioneering work in the field of treatment of xerostomia induced by drugs with topical acidic salivary stimulants.

The results of our clinical trial show that the use of 1% malic acid on spray causes an improvement of dry mouth feeling, and stimulates saliva production. Consequently, 1% malic acid may be an effective treatment of antihypertensive-induced xerostomia.
